# EMILIN2 (Elastin microfibril interface located protein), potential modifier of thrombosis

**DOI:** 10.1186/1477-9560-9-9

**Published:** 2011-05-11

**Authors:** Qila Sa, Jane L Hoover-Plow

**Affiliations:** 1 Joseph J. Jacobs Center For Thrombosis and Vascular Biology, Department of Cardiovascular Medicine and Department of Molecular Cardiology, Lerner Research Institute, Cleveland Clinic, Cleveland, OH 44195, USA

## Abstract

**Background:**

Elastin microfibril interface located protein 2 (EMILIN2) is an extracellular glycoprotein associated with cardiovascular development. While other EMILIN proteins are reported to play a role in elastogenesis and coagulation, little is known about EMILIN2 function in the cardiovascular system. The objective of this study was to determine whether EMILIN2 could play a role in thrombosis.

**Results:**

EMILIN2 mRNA was expressed in 8 wk old C57BL/6J mice in lung, heart, aorta and bone marrow, with the highest expression in bone marrow. In mouse cells, EMILIN2 mRNA expression in macrophages was higher than expression in endothelial cells and fibroblasts. EMILIN2 was identified with cells and extracellular matrix by immunohistochemistry in the carotid and aorta. After carotid ferric chloride injury, EMILIN2 was abundantly expressed in the thrombus and inhibition of EMILIN2 increased platelet de-aggregation after ADP-stimulated platelet aggregation.

**Conclusions:**

These results suggest EMILIN2 could play a role in thrombosis as a constituent of the vessel wall and/or a component of the thrombus.

## Background

The clinical manifestations of arterial and venous thrombosis represent the leading causes of death in the developed world [1]. While arterial and venous thrombosis have fundamental pathobiological differences, both are complex [2] and are influenced by multiple genetic and environmental factors [3]. Acute thrombosis at the site of a plaque is thought to be a precipitating event in the transition from a stable or subclinical atherosclerotic disease to acute myocardial infarction, ischemic stroke or peripheral arterial occlusion. For individuals undergoing surgery, thromboembolism and venous thrombosis are common. Twin and sibling studies [4] show that inherited risk factors contribute significantly to the development of coronary artery disease and ischemic stroke. Genetic abnormalities that influence production, activity, or metabolism can shift the balance in favor of thrombosis. Polymorphisms [2,5] in coagulation factors, fibrinolytic factors, platelet surface receptors, methylenetetrahydrofolate reductase, endothelial nitric oxide synthase and antioxidant enzymes have been implicated as genetic factors of risk for thrombosis. The role of many of these risk factors in thrombotic diseases has been replicated in animal models [6-11]. Great strides have been made in the diagnosis and treatment of thrombosis in the last decade. However, strategies to prevent thrombosis have lagged far behind, due in part to the contribution of multiple and as yet undefined genetic factors that lead to thrombotic risk. The objective of this study was to investigate whether EMILIN2 (elastin microfibril interface located protein 2), distributed in the cardiovascular system during development [12], plays a role in thrombosis.

**Figure 1:**
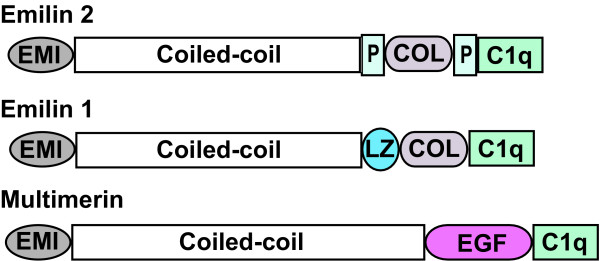
**EMILIN Protein Domains**. EMILIN2, EMILIN1 and Multimerin protein domains. Modified from Leimeister et al. [22] Grey: EMI (amino-terminal cysteine rich) domain; open: coiled-coil domain; light blue: P (proline-rich) domains; light grey: COL (collagen) domain, and green: C1q (complement component 1, subcomponent q) domain.

The EMILIN proteins are a group of extracellular matrix multimeric glycoproteins [13] including EMILIN1, Multimerin1, Multimerin2 and EMILIN2. The EMILIN proteins share four protein domains (Figure 1): C-terminal C1q domain, collagenous domain, coiled-coil domain and N-terminal cysteine-rich domain (EMI domain). The EMILIN proteins contain unique domains that are not shared: EMILIN1 has two leucine zipper regions; multimerin has an endothelial growth factor-like domain; and EMILIN2 contains a proline-rich domain. The domain organization suggests some shared and some specific functions for each of these EMILIN proteins. The proline-rich domain in EMILIN2 could provide structural flexibility and unique protein-protein interacting sites. EMILIN2 most closely resembles EMILIN1 [12], sharing 70% and 75% identity at the N- and C-terminal domains, respectively. Mouse EMILIN2 [12] has 73% identity with human EMILIN2.

While little is known about the function of EMILIN2, functions have been identified for EMILIN1 and Multimerin1. The C1q domain is homologous to C1q domains in a variety of proteins that are often involved in targeting proteins to cell surfaces and to other proteins [14]. C1q is the target recognition domain of the classical pathway of complement activation and a connecting link between the innate and acquired immunity systems [15]. The C1q domains bind to form homo- and hetero-multimers. EMILIN2 can form heterotrimers with EMILIN1 and partially co-localizes with EMILIN1 in cell culture. EMILIN1 deficient mice [16] are fertile, growth rate is similar to wild-type mice, and they show no gross abnormalities. However, they have hypertension, deficits in elastogenesis, and defects of the lymphatic vasculature [16,17]. In addition to its role in vascular elastic fiber structure, EMILIN1 promotes cell adhesion and/or cell migration of several soft tissue sarcomas and hematopoietic cells [12]. Studies have reported that the EMILIN1 C1q domain mediates cell adhesion through α4β1 integrin [18]. Unlike other members of the EMILIN family, Multimerin contains an RGD site and binds to αIIβ3 on platelets and αvβ3 on endothelial cells [19]. Cell adhesion to immobilized Multimerin1 involves RGD-dependent and -independent mechanisms, depending on the cell type. The binding of endothelial cells, activated neutrophils and smooth muscle cells to Multimerin is not RGD dependent. In addition, Multimerin1 [20] is found as a complex with platelet factor V in platelet α-granules and is released to the platelet surface upon platelet activation. It is also present in endothelial cells. The structural features of EMILIN1 and EMILIN2 suggest a unique, but related function for EMILIN2 in vascular homeostasis.

The major sites of expression of the *EMILIN *genes are in the cardiovascular system [21-23]. Other elastin associated binding proteins, including EMILIN1, fibulins and fibrillins [24,25] are important for the integrity of the vessel wall structure. EMILIN1 deficiency causes disruptions of the endothelial cell layer and interruptions of the elastic lamellae of large vessels [16]. Fibulin1 binds to elastin and fibrinogen and is incorporated into fibrin clots and atherosclerotic plaques [16]; fibulin1 deficiency causes hemorrhaging due to the abnormal ECM; fibulin 5 deficient mice have altered vascular remodeling [26] and fibulin4 deficient mice [26,27] are susceptible to aneurysm formation and aortic valve abnormalities. Hypertension is found in Emilin1 deficient mice and numerous studies indicate that hypertension is a risk factor for thrombosis. The association of these ECM proteins in the vascular wall with vascular remodeling after injury and hemostasis, implies that EMILIN2 may also share these functions and points to a potential role of EMILIN2 in thrombosis. The purpose of this study was to assess whether EMILIN2 plays a role in thrombosis.

The results of this study indicate that EMILIN2 does have the potential to regulate thrombosis. The major source of *Emilin2 *expression is the bone marrow. EMILIN2 is expressed in aorta, carotid artery wall, and ferric chloride-induced thrombi. In addition, inhibition of EMILIN2 causes de-aggregation of *in vitro *ADP-stimulated platelets. This study suggests EMILIN2 may be a modifier of thrombosis.

## Methods

### Mice

C57BL/6J mice (#000664) were obtained from Jackson Laboratory (Bar Harbor, Maine) at 6 weeks of age. Mice were housed at the Biological Resource Unit at the Cleveland Clinic Lerner Research Institute. Mice were tested between 7 and 9 weeks of age. All animal experiments were performed in accordance with a protocol approved by the Institutional Animal Care and Use Committee at the Cleveland Clinic.

### Cells and cell culture

The mouse cell lines, endothelial cells (EOMA), fibroblast cells (NIH3T3) and macrophage-like cells (Raw264.7) were purchased from the ATCC Global Biosource Centre (Manassas, VA). The cells were maintained according to the guidelines for each cell line in DMEM containing 10% FBS (Invitrogen, Carlsbad, CA) at 37°C with 5% CO_2 _and harvested at 90% confluence. Bone marrow was collected from the femurs and cells were harvested by centrifugation at 100×g for 10 min then washed with PBS.

### RT-PCR and real-time PCR

Total RNA was purified from the tissues and the cells using Qiagen RNeasy Mini Kit (Qiagen, Valencia, CA), treated with TURBO DNase (Ambion, Austin, TX) and reverse transcripted into cDNA using SuperScript™ III First-Strand Synthesis System for RT-PCR (Invitrogen, Carlsbad, CA) according to the manufacturer's instructions. RT-PCR was performed using 50 ng cDNA from mice as templates. The PCR product was analyzed on 1% or 1.2% agarose gel and recovered by Qiaquick Gel Extraction Kit (Qiagen). Sequencing (ABI 3730xl, Applied Biosystems, Foster City, CA) was performed at the Genomics Core of Cleveland Clinic. Negative controls that omit reverse transcription were included. Real-time PCR was performed using a BioRad iCycler iQ (BioRad, Hercules, CA). Each amplification reaction contained 20 ng of cDNA, 300 nM of each primer, 25 μL of 2× power SYBR Green Master Mix (Applied Biosystems, Warrington, UK), and 0.5 μL of UNG (Applied Biosystems) added to prevent carryovers. Samples were normalized to glyceraldehyde-3-phosphate dehydrogenase (GAPDH, Operon, Huntsville, AL). The comparative cycle threshold method was used to analyze the data. RNA was isolated from 5-6 mice for each group and analyzed in triplicates.

### FeCl_3 _carotid injury and immunohistochemical staining

To induce thrombus formation in the carotid artery, a ferric chloride (FeCl_3_) model of vessel injury was employed as previously described [28]. The flow probe (Model 0.5PSB, Transonic Systems, Ithaca, NY) was in place from baseline measurements to several minutes after the stable occlusion had been reached, or stopped at 30 min if it had not occluded. Blood flow was recorded every 10 sec (Model TS420, Transonic Systems). After cardiac perfusion, the carotid arteries were harvested after occlusion, immediately embedded into OCT (Tissue-Tek, Torrance, CA) and frozen. The frozen carotid arteries were sectioned at 10 μm using a cryostat (Leica CM1850, Leica Microsystems, Nassloch, Germany), fixed with acetone at 4°C for 10 min then blocked with normal serum. EMILIN2 was detected with E185 (rabbit anti-peptide antibody, see additional files 1 and 2) or Q-16 (goat antibody, sc-51356, Santa Cruz Biotechnology, CA), and P-selectin with rabbit anti-CD62P antibody (BD biosciences, San Diego, CA). The sections were then incubated with 1:1000 diluted biotinylated appropriate secondary antibodies (PK-6101, PK-6105 Vectastain ABC Kit, Vector Laboratories, Burlingame, CA) and proteins were visualized with alkaline phosphatase substrate. For quantification analysis, six sections of each carotid and six mice from each group were measured and analyzed using Image-Pro Plus (Media Cybernetics, Silver Spring, MD).

### Platelet aggregation assay

Blood was collected into sodium citrate from the vena cava of anesthetized mice. The platelet rich plasma (PRP) was collected by centrifugation at 100×g for 10 min, and cell pellets were centrifuged at 1000×g for 10 minutes to collect platelet poor plasma (PPP). Platelets were counted using a Cellometer Auto M10 (Nexcelom Bioscience, Lawrence, MA) and adjusted with PPP to 2-3×10^8^/ml. PRP was incubated with 26 μg/ml anti-EMILIN2 antibody E185, or at a concentration as indicated, or control antibody (Calbiochem, La Jolla, CA) for 3 minutes at 37°C followed by the addition of 20 mM ADP (Chrono-log Corporation, Havertown, PA). Platelet aggregation was measured in an aggregometer (Chrono-log Corporation) and each sample was allowed to run for 8 minutes with stirring at 37°C.

### Statistical analysis

Differences between strains were determined by a t-test or ANOVA with a Newman-Kuels Multiple Comparison post-test. Data are presented as mean ± SEM.

## Results

### Distribution of *Emilin2 *mRNA in tissues

**Figure 2:**
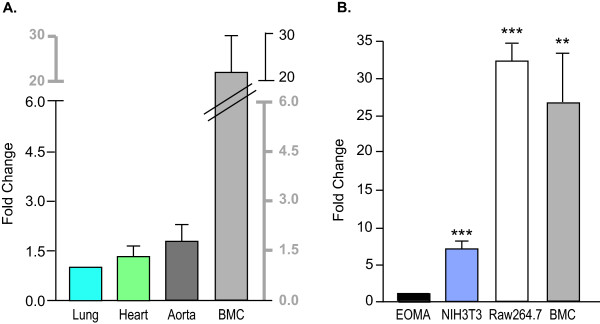
***Emilin2 *mRNA Expression in Tissues and Mouse Cells**. GAPDH from the same cDNA samples used as endogenous control. **A**. Expression of *Emilin2 *in lung, heart, aorta and bone marrow cells was determined by real-time PCR. Aorta, n = 3; others, n = 5. The mRNA levels are presented as fold change relative to lung. The values are mean ± SEM n = 3-5. **B**. Expression of *Emilin2 *in mouse cell lines. The mRNA levels are presented as fold change relative to EOMA. EOMA, mouse endothelial cell line; NIH3T3, mouse fibroblast cell line; Raw264.7, mouse macrophage-like cell line. The values are mean ± SEM of 3 independent experiments. Statistical differences compared to EOMA were determined by ANOVA and Newman-Kuels post-test. **P < 0.01, ***P < 0.001.

The mRNA expression of *Emilin2 *was determined by quantitative real-time PCR in lung, heart, aorta and bone marrow cells (Figure 2A). The expression in the bone marrow was 20-fold higher than in the lung. This suggests that bone marrow is a major source of *Emilin2 *and is unlike *Emilin1 *expression [21] where the major source is the vessel wall.

Expression of *Emilin2 *was tested in a mouse macrophage-like cell line (Raw264.7), and two other cell types associated with vascular wall, a mouse endothelial cell line (EOMA), and a mouse fibroblast cell line (NIH3T3). The relative expression in macrophage cells was comparable to expression in mouse bone marrow cells (Figure 2B). The expression of *Emilin2 *was 7-, 32- and 26-fold higher in NIH3T3, Raw264.7 and bone marrow cells, respectively, compared to EOMA cells suggesting that the expression of *Emilin*2 may be high in hematopoietic progenitor stem cells, precursors for white and red blood cells and platelets.

### EMILIN2 was expressed in the vessel wall and in the thrombus induced by vascular injury

**Figure 3:**
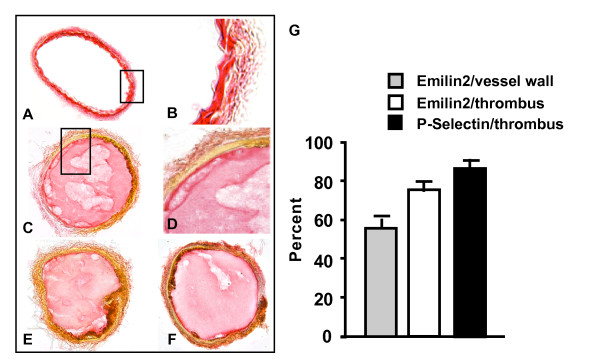
**Localization of EMILIN2 in carotids**. **A**, **B**. Sections from uninjured carotid were immunostained with E185 for EMILIN2. **C**, **D**, **E**. Ferric chloride induced vascular injury. Sections were immunostained with C. E185, D. Q-16, or F. P-selectin antibody. **A**, **C**, **E**, **F**. Magnification × 100 and B, D. × 400. G. Quantification of immunostaining. The values are mean ± SEM of the percent of EMILIN2 (E185)/vessel wall, EMILIN2 (E185)/thrombus area and P-selectin/thrombus area of 6 mice per strain. Six sections per mouse were analyzed for each mouse using Image-Pro Plus software. Modified from Sa et al [Mamm Genome 2010 21:337-349].

EMILIN2 was detected in the vessel wall of uninjured carotids (Figure 3A, B). The immunostaining was 55% ± 2 of the vessel wall area (Figure 3G, gray bar). EMILIN2 was detected in both cells and extracellular matrix. The aorta clearly defines the sites of Emilin2 (see additional file 3). To determine if EMILIN2 protein is incorporated into the thrombus, FeCl_3 _was used to induce thrombus formation in the carotid artery. After injury, carotid arteries were harvested and immunostained for EMILIN2 (Figure 3C, D). Strong (77% ± 2) (Figure 3G open bar) EMILIN2-specific staining was detected in thrombi, suggesting that EMILIN2 is associated most strongly with the thrombus rather than the vessel wall. This is consistent with the mRNA expression in the bone marrow cells, platelet, and plasma protein expression. No signal was detected in the sections stained with rabbit IgG or pre-immune rabbit serum. The staining of EMILIN2 in thrombi was confirmed by using the commercial EMILIN2 Q-16 antibody (Figure 3E).

The high expression of EMILIN2 in bone marrow and in the thrombi raised the question whether EMILIN2 was involved in platelet function. Therefore, carotid sections were also immunostained for P-selectin (Figure 3F), one of the platelet markers. Strong immunostaining (Figure 3G, black bar) for P-selectin (88% ± 1 of thrombi area) revealed a similar distribution pattern to that found for EMILIN2 (77% of thrombi area) in the injured carotids.

### EMILIN2 antibody inhibits platelet aggregation

**Figure 4:**
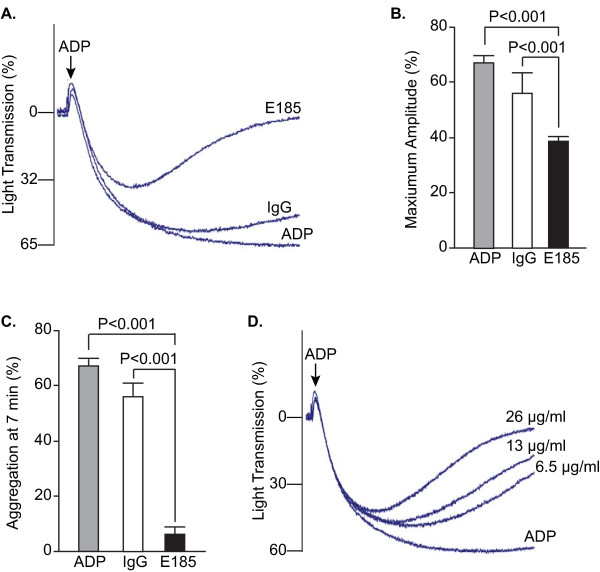
**EMILIN2 antibody inhibited mouse platelet aggregation**. **A**, **B**, **C**. Platelet aggregation in platelet-rich plasma was induced by ADP and optically measured with an aggregometer. Platelets were pre-incubated with affinity purified rabbit anti-mouse EMILIN2 antibody (E185) or rabbit IgG as a control. A. Representative aggregation curves are shown. **B**. Quantification of aggregation results expressed as maximal amplitude of aggregation (mean ± SEM, n = 3-6). **C**. Quantification of platelet aggregation rate at 7 minutes after addition of ADP (mean ± SEM, n = 3-6). **D**. Representative platelet aggregation curves in platelet-rich plasma from B6-Chr17^A/J ^mice. Concentrations of EMILIN2 antibody are indicated. Statistical differences between ADP or IgG and E185 were determined by ANOVA with a Newman-Kuels Multiple Comparison Test.

The similar relative distribution of EMILIN2 and platelets in the thrombi suggested that EMILIN2 may regulate clot stability through a role in platelet aggregation. Pre-incubation of platelets with anti-EMILIN2 antibody inhibited the extent and rate of platelet aggregation induced by ADP (Figure 4A). Pre-incubation of platelets with EMILIN2 antibody inhibited the maximum amplitude of platelet aggregation by approximately 42% (Figure 4B). Moreover, the EMILIN2 antibody caused platelet de-aggregation in the presence of high ADP concentrations (20 μM) over induction time (Figure 4C). At 7 minutes after addition of 20 μM ADP, platelet de-aggregation in the presence of the antibody was inhibited by 90%. Inhibition of ADP-induced platelet aggregation by EMILIN2 antibody was dose dependent, with the highest inhibition achieved at 26 μg/ml (Figure 4D). These data suggest that EMILIN2 functions in stabilizing aggregated platelets.

## Discussion

In this study we report evidence that EMILIN2 may play a role in thrombosis. EMILIN2 mRNA expression is high in bone marrow and higher in macrophages than endothelial cells or fibroblasts The protein distribution of EMILIN2 in thrombi was similar to P-selectin, a marker for platelets suggesting EMILIN2 may be associated with platelets. Further, EMILIN2 antibody increased platelet de-aggregation. EMILIN2 could play a role in thrombosis by maintaining the vessel wall architecture as a component of vessel wall or in the regulation of the platelet aggregation stability by virtue of its presence in plasma and platelets. This is the first report of *in vivo *protein expression of EMILIN2 in adult tissues and to provide evidence for a potential role of EMILIN2 in thrombosis.

While EMILIN2 has a high homology to EMILIN1 and the molecular structures of EMILIN1 and EMILIN2 are similar, there are distinctive molecular differences between the two proteins. In addition, the pattern of mRNA expression in embryonic development [21] is different between EMILIN1 and EMILIN2 and the expression pattern is also different in adults [21]. The differences in molecular structure and expression suggest the proteins have different functions. On the other hand, there are similarities as well. EMILIN2 and EMILIN1 are extracellular matrix proteins and are found in the vasculature. EMILIN1 deficiency disrupts the structure of the vascular wall and impairs elastogenesis. A deficiency of other microfibril and elastin associated molecules also disrupt elastogenesis, including fibrillin-1, fibrillin-2, microfibril associated glycoprotein 1 (MAGP1) [29] and the fibulins 1-5 [21-26,30,31]. Fibulin 4 heterozygous mice (the null mutation is lethal) have prolonged clot stability in the tail-bleeding/rebleeding assay (Hoover-Plow, J and Chen, Q, unpublished results). MAGP1 deficient mice have delayed thrombotic occlusion after vascular injury [29]. To determine whether EMILIN2 plays an important role in vascular structure and response to vascular injury will require the construction of genetically modified mice.

Platelet may be a site producing or storing EMILIN2. Multimerin1, another EMILIN protein, is stored in the platelet alpha granules bound to Factor V and is released upon platelet activation [20]. EMILIN2 could be a component of platelet alpha granules or alternatively, plasma EMILIN2 could bind to the platelets and exert an effect on thrombus stability. Factor V, a cofactor needed for thrombin generation, is present in both plasma and platelets and may be taken up by the platelets [19].

All adult tissues that have been tested contain EMILIN2, cochlear basement membrane [32], brain, spleen, liver [33] and heart [34,35]. This would be expected of an ECM protein. In addition, a number of proteomic and microarray studies of human tissues (Table 1) have identified EMILIN2 in extracellular fluids (plasma, synovial, amniotic and seminal plasma), stem cells (hematopoietic, mesenchymal, osteoblasts), stimulated endometrial stromal cells, cancer cells (hepatic, lymphocytic leukemia, colorectal cancer, and ovarian cancer), and as a cell surface marker for human embryonic stem cell-derived cardiomyocytes [36-50]. A recent study [40] reported down-regulation of EMILIN2 in brain arteriovenous malformations. These studies suggest EMILIN2 plays important physiological and pathological roles. The identification of EMILIN2 in plasma in humans as well as a component of mouse thrombus supports the hypothesis that EMILIN2 could play a role in thrombosis.

**Table 1:** EMILIN2 expressed in human tissues

Tissue	Reference
Plasma/serum^	[36]
Amniotic fluid^	[37]
Seminal plasma^	[38]
RA synovial fluid^	[39]
Decreased expression in brain arteriovenous malformations*	[40]
Increased in granulocytes after G-CSF treatment*	[41]
Increased in mesenchymal stem cells*	[42]
Osteoblast ECM matrix vesicles^	[43]
Decreased in endometrial stromal cells treated with cAMP*	[44]
Hepatic carcinoma cells*	[45]
Hepatic carcinoma cells^	[46]
Chronic lymphocytic leukemia cells*	[47]
Colorectal cancer nuclear matrix^	[48]
Fragments in sera of patients with ovarian cancer^	[49,50]
Human embryonic stem cell-derived cardiomyocytes^	[34]

^ - protein expression; * - mRNA expression

## Conclusions

In this study, we found EMILIN2 expression in mouse lung, heart, aorta, and bone marrow. Protein expression in the carotid vessel wall and thrombi suggesting EMILIN2 could function directly in the thrombus and/or as a constituent of the vessel wall. EMILIN2 was identified in humans in plasma/serum, amniotic fluid, seminal plasma, stem cells, cancer cells, and heart stem cells suggesting EMILIN2 may play important physiological/pathological roles. Further studies, are needed to identify the role of EMILIN2 in platelet aggregation.

## Competing interests

The authors declare that they have no competing interests.

## Authors' contributions

QS performed experiments, analyzed data, and participated in writing the paper. JHP designed and planned the study, analyzed data, and wrote the paper. Both authors have read and approved the final manuscript.

## Supplementary Material

Additional file 1 **Figure 1: EMILIN2 peptide for antibody generation.** In order to measure EMILIN2 protein and to study its functions, a polyclonal antibody for mouse EMILIN2 was generated in rabbits. Since EMILIN2 has a high homology to EMILIN1, a peptide was synthesized from residues 829-843 located in the proline-rich domain specific to EMILIN2 (see additional file 1). To increase the specificity of the antibody, the rabbit antiserum was subjected to affinity-purification using the peptide. Click here for file

Additional file 2 **Figure 2: EMILIN2 antibody specificity.** To test the specificity of the antibody, HEK293 cells were transfected with the construct E2-N1 that contains the EMILIN2-EGFP fusion gene and construct PR-N1 that harbors the PR domain-EGFP fusion gene. The vector N1 that contains only the EGFP gene was used as negative control. Constructs containing EMILIN2 protein domains, namely Collagen-like C1q domains that do not interact with the antibody, and EGFP fusion genes, were also used as negative controls. Click here for file

Additional file 3 **Figure 3: EMILIN2 Immunostaining of aorta**. After cardiac perfusion, the aortas were harvested, immediately embedded into OCT (Tissue-Tek, Torrance, CA) and frozen. The frozen aortas were sectioned at 10 μm using a cryostat (Leica CM1850, Leica Microsystems, Nassloch, Germany), fixed with acetone at 4°C for 10 min then blocked with normal serum. EMILIN2 was detected with E185 antibody. The section was then incubated with 1:1000 diluted biotinylated appropriate secondary antibodies (PK-6101, PK-6105 Vectastain ABC Kit, Vector Laboratories, Burlingame, CA) and EMILIN2 visualized (brown color) with alkaline phosphatase substrate. Click here for file
